# Risk of Urinary Bladder Cancer in Patients With Inflammatory Bowel Diseases: A Meta-Analysis

**DOI:** 10.3389/fsurg.2021.636791

**Published:** 2021-05-26

**Authors:** Zhihua Geng, Qing Geng

**Affiliations:** ^1^Department of Orthopaedics, Union Hospital, Tongji Medical College, Huazhong University of Science and Technology, Wuhan, China; ^2^Department of Thoracic Surgery, Renmin Hospital of Wuhan University, Wuhan, China

**Keywords:** bladder cancer, Crohn's disease, inflammatory bowel diseases, ulcerative colitis, meta-analysis

## Abstract

A systematic search of the PubMed, Cochrane, Embase, and Web of Science databases was conducted to investigate the risk of urinary bladder cancer (BC) in patients with inflammatory bowel disease (IBD). We identified 168 articles, of which 11 met the inclusion and exclusion criteria. Our analysis included 165,176 patients with IBD, 491 of whom had BC. Overall, the pooled standardized incidence ratio (SIR) was 0.99 (95% CI: 0.87–1.12; *I*^2^ = 0%). Further subgroup analysis showed that BC risk was neither statistically higher for Crohn's disease (CD) (SIR: 1.19; 95% CI: 0.94–1.44; *I*^2^ = 0%) nor for patients with ulcerative colitis (UC) (SIR: 0.92; 95% CI: 0.77–1.06; *I*^2^ = 0%). In the analysis of two case-control studies providing data on BC in UC and CD combined, IBD patients seemed to have a higher risk of BC than non-IBD patients (relative risk: 1.25; 95% CI: 0.77–2.03; *I*^2^ = 37.5%). Although the overall risk of BC was not significantly increased among patients with IBD, there was a weak trend for the risk to be elevated in CD patients, indicating marginal significance. These findings may primarily be explained by the opposite effects of smoking on CD and UC as well as the immunosuppressive drugs these patients often take.

## Introduction

Inflammatory bowel diseases (IBDs), including Crohn's disease (CD) and ulcerative colitis (UC), are chronic idiopathic disorders causing inflammation of the gastrointestinal tract (1). The number of individuals affected by IBD remains unclear. However, estimates suggest that more than 1.5 million and 2 million people are suffering from the disease in North America and Europe, respectively, with a rapidly growing number in Asia and other newly industrialized areas, placing heavy burdens on the health systems of countries in these areas (2, 3). IBD is characterized by recurrent mucosal inflammation, bowel obstruction resulting from intestinal strictures, and internal or external fistulas or intestinal perforation (1). Moreover, IBD could involve extraintestinal organs and lead to corresponding manifestations. The most common extraintestinal manifestations include rheumatologic disorders, dermatologic disorders, ophthalmologic disorders, primary sclerosing cholangitis, nephrolithiasis, and thromboembolic events (4).

Increasing evidence shows that a fair number of human cancers are caused by chronic infection or chronic inflammatory states (5). Not surprisingly, IBD, with a continuous inflammatory state in the intestine, increases the risk of intestinal tumors in these patients. IBD patients are two to six times more likely to develop colorectal cancer (CRC) than the general population, and they tend to be younger than sporadic CRC patients (6). More importantly, patients with CD and UC are at increased risk of both intestinal and extraintestinal malignancies, and they have a higher risk of developing invasive cancer than the general population, including lymphoma, melanoma, and cholangiocarcinoma (7). However, there is a lack of conclusive evidence demonstrating the risk of urinary bladder cancer (BC), the ninth most common cancer around the world, in IBD patients (8, 9). Here, we carried out a comprehensive meta-analysis to investigate the relationship between IBD and the risk of BC.

## Methods

### Literature Search

To identify population-based papers on the risk of BC in patients with IBD, we conducted a systematic search for articles from inception to November 2020 in PubMed, Cochrane, EMBASE, and Web of Science. The full search strategies used for each database are described in the Supplementary File. The systematic search and data extraction were performed using the Preferred Reporting Items for Systematic Reviews and Meta-Analyses (PRISMA) guidelines. Ethical approval and patient consent are not required for meta-analyses. All potentially eligible studies were scrutinized regardless of their primary outcomes or language, and references from related articles were also retrieved manually to ensure a comprehensive search.

### Inclusion and Exclusion Criteria

Eligible studies included in the meta-analysis had to meet the following criteria: (1) population-based studies involving valid diagnostic criteria for CD and/or UC (10); (2) patients developed BC after being diagnosed with IBD, either UC or CD; (3) a sample size >1,000; (4) the association between IBD and the risk of BC was evaluated using standardized incidence ratio (SIR) or relative risk (RR) with 95% confidence intervals (CIs); (5) population-based cohort or case-control studies; and (6) a follow-up of more than 1 year. Studies that met the following criteria were excluded: (1) meta-analyses or review articles; (2) UC and CD were not distinguished effectively in the article; (3) relevant data were not available; and (4) repeated literature or republished. If the study population overlapped, the most recently published study or most informative study was included. Two authors independently screened the title and abstract according to these eligibility criteria, screened the full text articles against the inclusion criteria, conducted data extraction, and performed a quality evaluation. In cases of discrepancies, consensus was reached through a discussion with a senior researcher.

### Data Extraction and Bias Assessment

The data from the eligible studies were extracted by two independent researchers using a predefined data extraction form: the name of the first author, year of publication, sample size, study design, period, country, cancer types, and outcomes (SIR or RR). When disagreements arose, a consensus was reached through discussions with a senior researcher.

The Newcastle-Ottawa Scale (NOS) was used to evaluate the methodological biases of the included studies (11). The NOS uses a “star system” to assess the quality of each study from three perspectives: the selection of the study groups, the comparability of the groups, and an assessment of the outcomes. If seven or more of the possible total of nine stars is received, the study is considered high quality. Moreover, the level of evidence (LoE) of the included articles was rated by two independent researchers using the Oxford Centre for Evidence-Based Medicine criteria, which grades studies from the strongest (level 1) to the weakest (level 5) strength of evidence on the basis of the study design and data quality (12).

### Statistical Analysis

Pooled risk estimates (SIR, observed/expected) with 95% confidence intervals (CIs) for BC were calculated using the STATA version 14.2 meta-analysis program (Stata Corporation, College Station, TX, USA). The Q and *I*^2^ statistics were calculated to estimate the heterogeneity among studies. If Q tests' *p* > 0.1 or *I*^2^ ≤ 50%, fixed-effects models were applied. Otherwise, a random-effects model was used. Funnel plots were used to assess the possibility of publication bias, and the asymmetry of the funnel plots was evaluated by Egger's test. Significant publication bias was defined as a *p* < 0.1. Sensitivity analyses were applied to evaluate the robustness of the pooled results with a significance level of 5%.

## Results

### Characteristics of the Study Subjects

**Figure 1:**
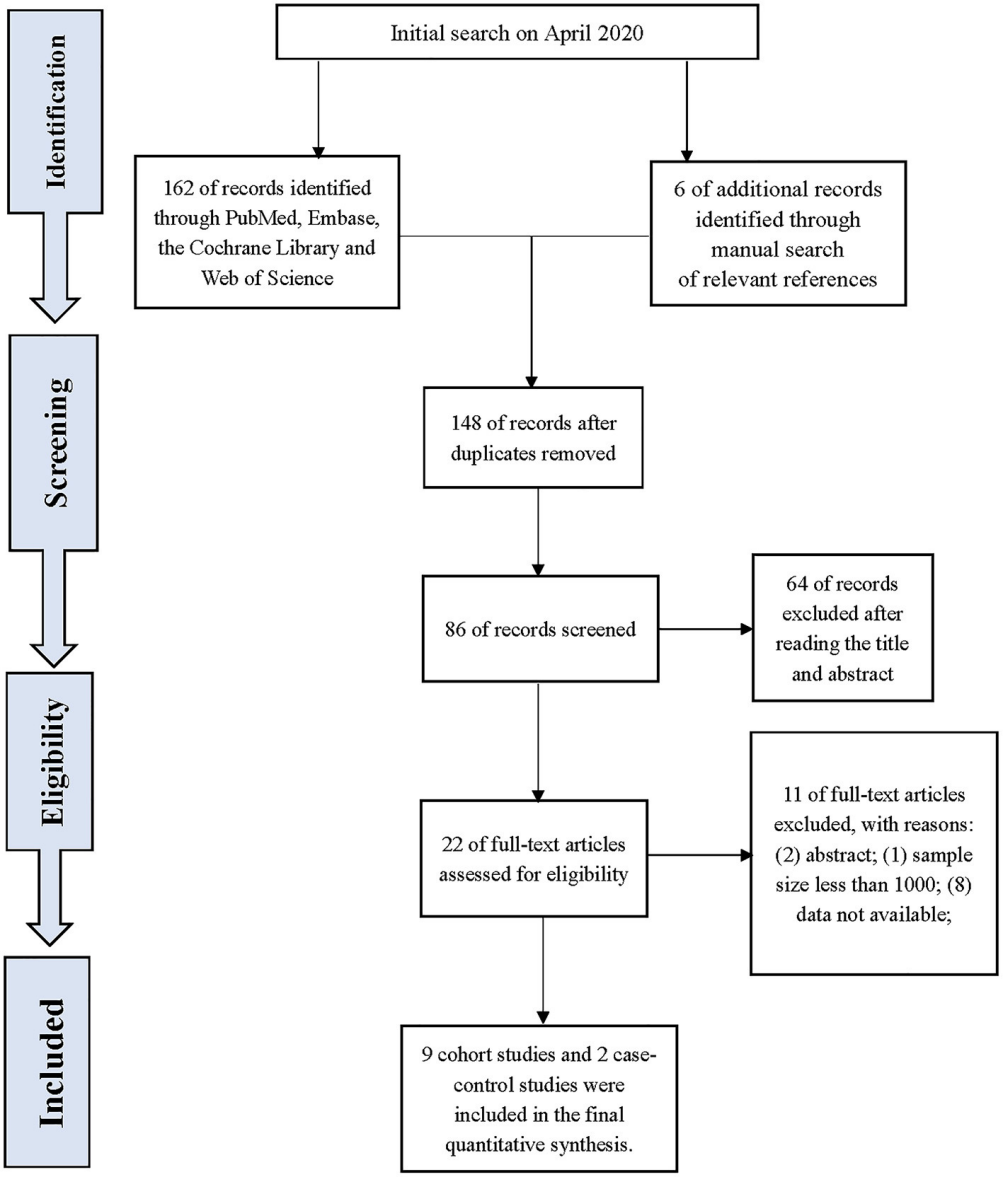
Flow diagram of searching, inclusion, and exclusion of relevant studies.

Our search identified 168 studies. After reviewing and screening, 11 studies were finally included in the review of the full text, including nine cohort studies and two case-control studies. The study flow diagram is presented in Figure 1. A total of 442, 603 participants were involved in this population-based study with 157, 069 from cohort studies and 285, 534 from case-control studies. The details of the included studies are listed in Table 1.

**Table 1:** Characteristics of included literature on bladder cancer in patients with inflammatory bowel disease.

**Study**	**Country**	**Design**	**Period**	**Population**	**Outcomes**	**NOS**	**LoE**
Hemminki et al. (13)	Sweden	Cohort	1964–2004	21788 CD	SIR	9	2b
Jung et al. (14)	Korea	Cohort	2011–2014	3918 CD and 5825 UC	SIR	8	2b
Jussila et al. (15)	Finland	Cohort	2000–2010	5315 CD and 16649 UC	SIR	7	2b
Kappelman et al. (7)	Denmark	Cohort	1978–2010	13756 CD and 35152 UC	SIR	9	2b
Ekbom et al. (16)	Sweden	Cohort	1965–1983	1655 CD and 3121 UC	SIR	7	2b
Hemminki et al. (17)	Sweden	Cohort	1964–2004	27606 UC	SIR	9	2b
Karlen et al. (18)	Sweden	Cohort	1955–1989	1547 UC	SIR	8	2b
Bourrier et al. (19)	France	Cohort	2004–2005	11759 CD and 7727 UC	SIR	9	2b
Persson et al. (20)	Sweden	Cohort	1955–1989	1251 CD	SIR	7	2b
Mosher et al. (21)	USA	Case-control	1996–2015	2080 IBD and 271898 IBD-free	RR	7	3b
Bernstein et al. (22)	Canada	Case-control	1984–1997	6027 IBD and 5529 IBD-free	IRR	9	3b

*CD, Crohn's disease; UC, ulcerative colitis; SIR, standardized incidence ratios; NOS, Newcastle-Ottawa Scale; LoE, level of evidence*.

### Meta-Analysis Results

**Figure 2:**
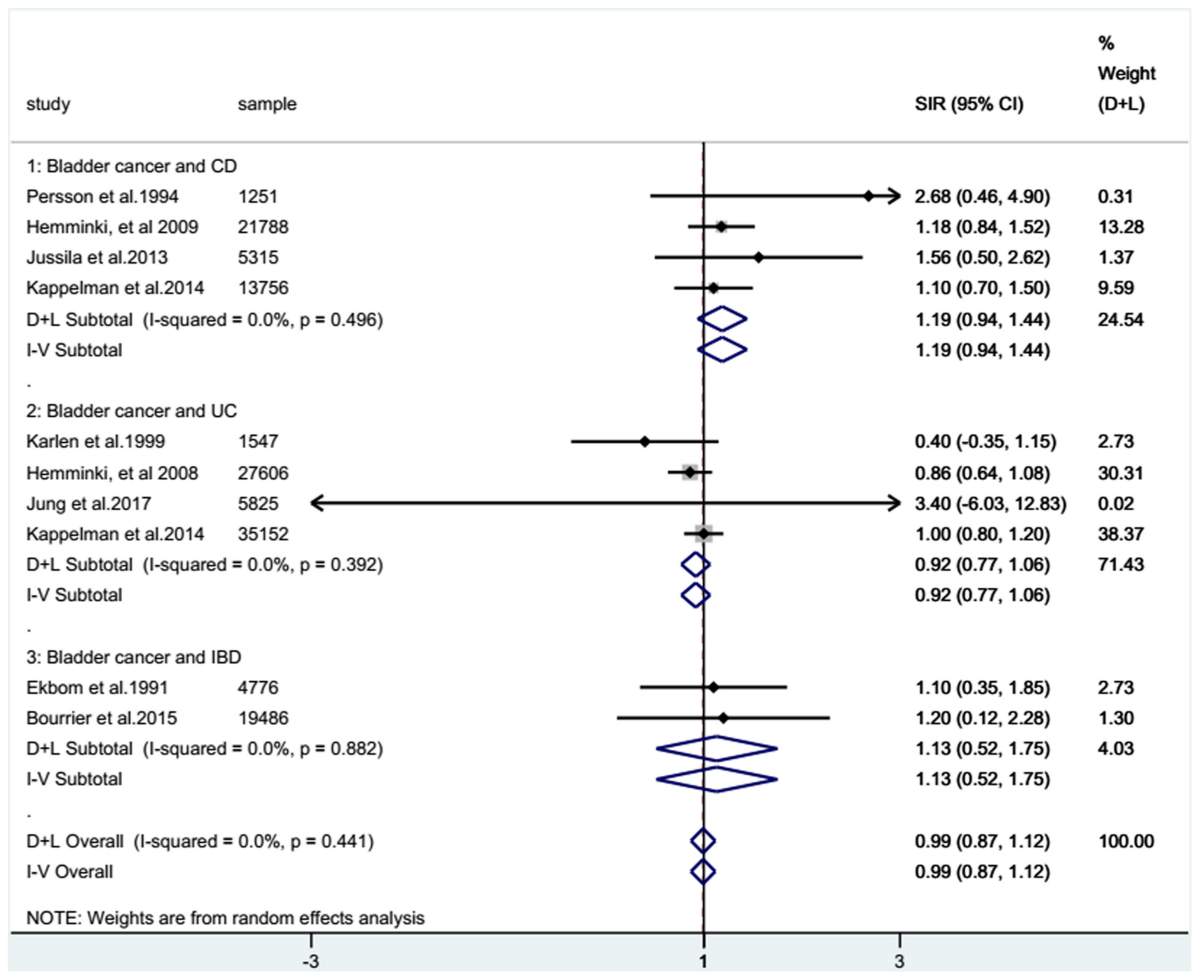
Combined standardized incidence ratios (SIRs) of all eligible cohort studies. Forest plots showing the combined SIRs with 95% confidence intervals for bladder cancer occurrence in inflammatory bowel disease patients, based on eligible population-based cohort studies. The size of the black boxes is proportional to the weight of the corresponding studies.

**Figure 3:**
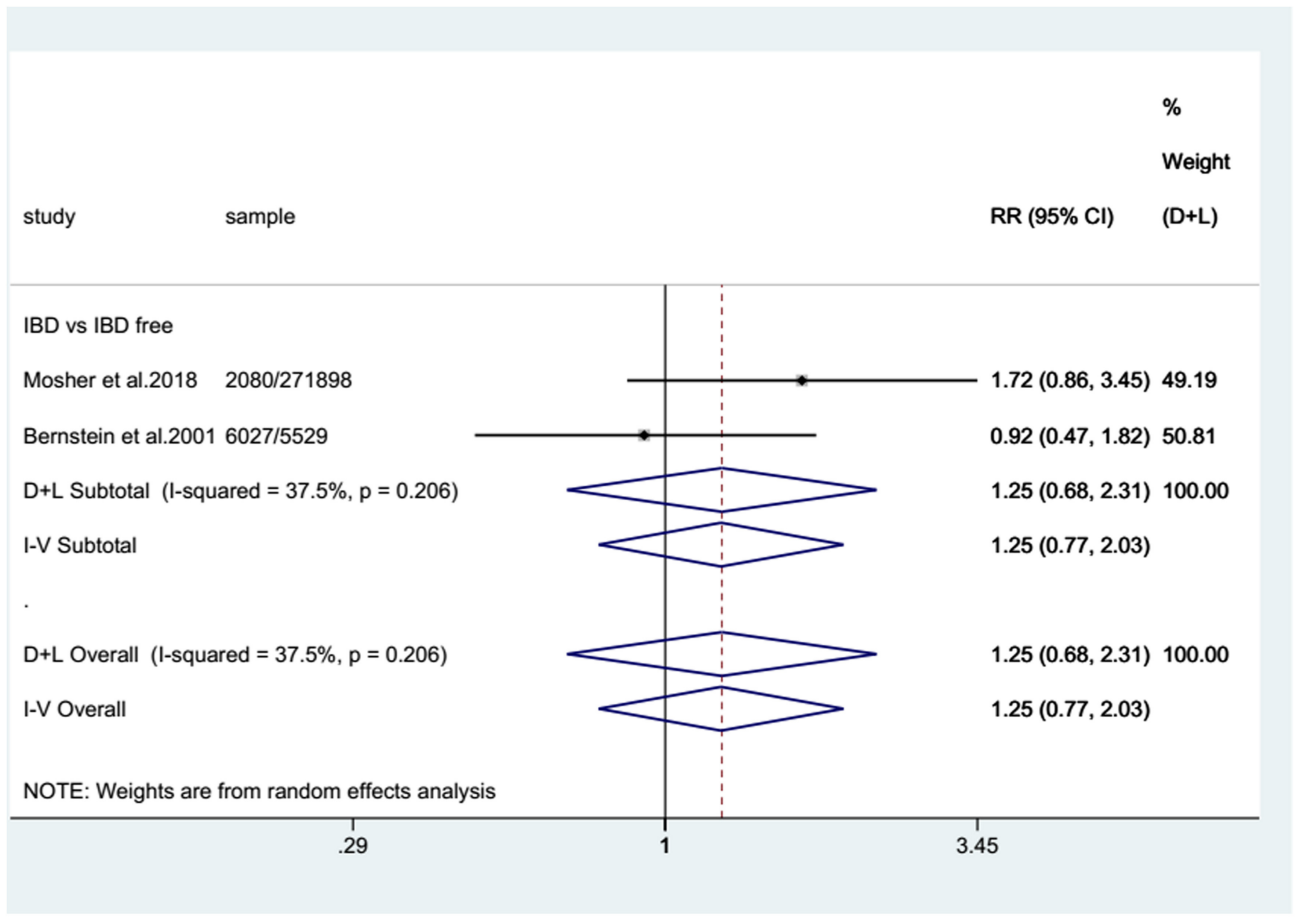
Pooled relative risks (RRs) of eligible case-control studies. Forest plot showing the pooled RRs with 95% confidence intervals for bladder cancer occurrence in inflammatory bowel disease patients from the two eligible case-control studies. The size of the black boxes is proportional to the weight of the corresponding studies.

For the cohort studies, no significantly increased risk of BC was observed in patients with IBD (SIR: 0.99; 95% CI: 0.87–1.12; *I*^2^ = 0%; Figure 2) or in the UC subgroup (SIR: 0.92; 95% CI: 0.77–1.06; *I*^2^ = 0%; Figure 2), whereas a trend toward an increased risk of BC was detected in patients with CD (SIR: 1.19; 95% CI: 0.94–1.44; *I*^2^ = 0%; Figure 2). There were two case-control studies that provided data on BC in UC and CD combined, revealing that IBD patients seemed to have a higher risk of BC than non-IBD patients (RR: 1.25; 95% CI: 0.77–2.03; *I*^2^ = 37.5%; Figure 3).

### Bias Assessment and Sensitivity Analyses

**Figure 4:**
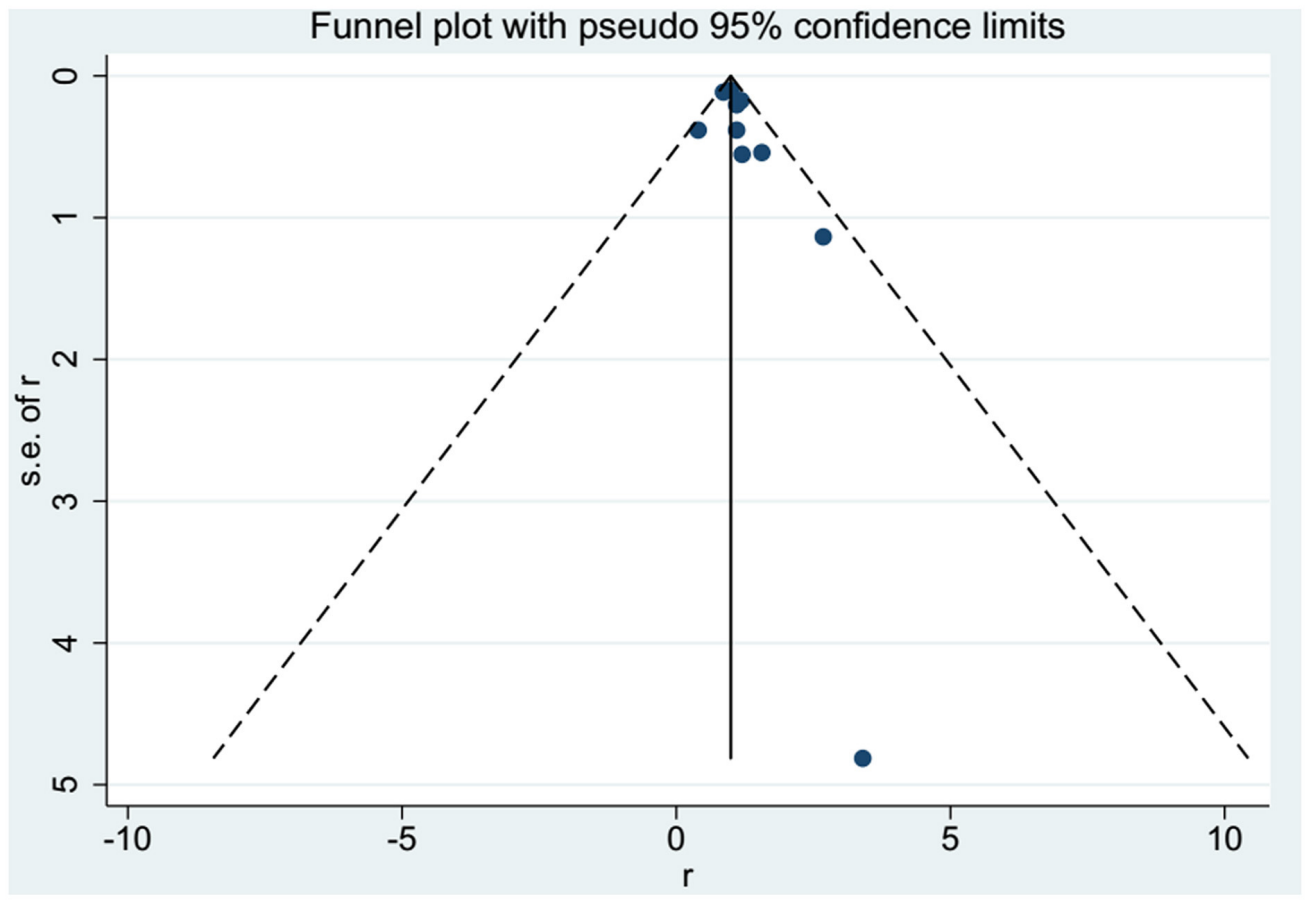
Funnel plot for eligible studies with pseudo 95% confidence limits.

No significant asymmetry was observed in the present study through the corresponding funnel plots (Figure 4). The *p*-value of Egger's test was 0.194. Furthermore, sensitivity analysis revealed that there was no significant change in the pooled results for all outcomes when excluding each study from the meta-analysis sequentially.

## Discussion

In the present meta-analysis, patients with IBD were not significantly associated with an increased BC risk. In the UC subgroup, no remarkable association was observed. However, in the CD subgroup, a trend toward an increased risk of BC was evident, indicating marginal significance.

IBD-related malignancies have been investigated by researchers in recent decades. The mechanism underlying inflammation-related carcinogenesis is mainly attributed to epigenetic alterations and DNA damage induced by inflammation as the key factors in carcinogenesis (23). Malignancies, both gastrointestinal and extraintestinal, are long-term complications in patients with IBD; they have a 30 and 10% higher long-term risk of invasive cancer than the general population, respectively, which might be a result of chronic inflammation and their use of immunosuppressive medications aimed at controlling inflammation (7, 8). It is widely accepted that chronic local inflammation in the bowel is involved in the carcinogenesis of CRC (24) although disputes still exist on whether the risk of CRC is increased in IBD patients (6, 7, 25, 26).

For extraintestinal cancers, previous epidemiological studies revealed a tight association between IBD and an increased risk of numerous cancers, such as hematologic cancer, lung cancer, and non-Hodgkin lymphoma (27). In addition, patients with IBD have a higher risk of tumors derived from the prostate, skin, liver, and biliary system (4). They were also proven to be at higher risk of developing BC in some single-center studies, both retrospectively and prospectively (8, 9). However, the risk of BC in those patients has not yet been conclusively corroborated by the latest research.

BC, as the ninth most common cancer worldwide with a yearly incidence of approximately 430,000 cases, ranks 13th in terms of yearly mortality from cancer with a male predominance, and it is the seventh most common cancer worldwide in men (28). The risk of BC in IBD patients has not been conclusively substantiated although IBD has been proven to be correlated with increased risks of several extraintestinal cancers (7). With respect to the risk of BC in IBD patients, the study by Kappelman et al. reveals that its risk was only marginally increased in CD patients with an SIR of 1.2 (95% CI: 0.8–1.6) (7). This is, in general, consistent with our result (SIR: 1.19; 95% CI: 0.94–1.44).

Additionally, the study of Kappelman et al. indicates that an increased risk of smoking-related malignancies was substantiated in these patients (7), which is consistent with cigarette smoking being thought to increase the risk of CD (29, 30). Otherwise, the studies of Madanchi et al. and Algaba et al. both suggest a higher risk of BC in IBD patients than that of the non-IBD population; the incidence of BC was 21.7/100,000 in the former study, 9.6/100,000 globally in men, and the RR of BC was 5.23 (95% CI: 1.95–13.87) in the latter study (8, 9, 31). Their conclusions were different from ours, which might be attributed to the different proportions of CD and UC patients in the various studies.

In the study of Natalia et al., the SIR of BC in patients with CD was 2.03 (95% CI: 1.14–3.63) and that in patients with UC was only 0.55 (95% CI: 0.26–1.16); collectively, their study shows that the SIR of IBD patients was only 0.99 (95% CI: 0.63–1.54), indicating no difference between those patients and the general population (4). Our finding of a non-significant difference in BC risk is in accordance with the results produced by their analysis.

In our study, although the association of BC with UC and CD combined was weak, the SIR of BC in the CD subgroup was somewhat higher than that in the UC subgroup. This might be explained by the high prevalence of smokers among CD patients (32). Cigarette smoking is thought to increase the risk of CD and exacerbate the disease course; however, it plays an opposite role in UC, protecting against intestinal inflammation and improving its course (29, 30). Additionally, the main risk factor for BC is cigarette smoking; the trends and patterns of its incidence are closely correlated with the prevalence of smoking (28). Thus, a higher proportion of smokers among CD patients may account for their higher incidence of smoking-associated tumors, such as BC and other IBD-related malignancies. In addition, some studies report that long-term use of immunosuppressive medications in IBD patients increases the risk of cancer (33, 34). Moreover, immunosuppressant medications, which are widely used for IBD treatment, are believed to be responsible for elevated risk of cancers such as non-Hodgkin lymphoma, acute myeloid leukemia, non-melanoma skin cancers, and urinary tract cancers (19, 35–37). However, according to Ariela Holmer et al., there is limited data on the risk of malignancy with newer non-TNF-targeting biologics, including vedolizumab and ustekinumab or targeted small molecules, such as tofacitinib, in patients with IBD (38).

### Limitations

There are some limitations of this study. The included studies were mainly carried out in Western countries with a lack of research from other parts of the world. With the quick industrialization of Asian countries and other places, the incidence of IBD is increasing quickly in these areas. The correlations of BC and IBD in these areas have yet to be verified, and our conclusions may not be applicable for patients from those parts of the world.

## Conclusions

In conclusion, our meta-analysis suggested that the overall risk of BC in IBD patients and the risk of BC in UC patients are not increased significantly. However, the risk of BC in CD patients may be increased with marginal significance. These findings might be affected by different prevalences of cigarette smoking in the different populations and the use of immunosuppressive medications by these patients.

## Data Availability Statement

The raw data supporting the conclusions of this article will be made available by the authors, without undue reservation.

## Author Contributions

ZG: study design, data collections, data analysis, and drafting. ZG and QG: revising. QG: funding. Both authors contributed to the article and approved the submitted version.

## Conflict of Interest

The authors declare that the research was conducted in the absence of any commercial or financial relationships that could be construed as a potential conflict of interest.
