# Corrigendum: Risk of urinary bladder cancer in patients with inflammatory bowel diseases: A meta-analysis

**DOI:** 10.3389/fsurg.2022.1052518

**Published:** 2022-10-13

**Authors:** Zhihua Geng, Qing Geng

**Affiliations:** ^1^ Department of Orthopaedics, Union Hospital, Tongji Medical College, Huazhong University of Science and Technology, Wuhan, China; ^2^ Department of Thoracic Surgery, Renmin Hospital of Wuhan University, Wuhan, China

**Keywords:** bladder cancer, crohn's disease, inflammatory bowel diseases, ulcerative colitis, meta-analysis

A corrigendum on Risk of urinary bladder cancer in patients with inflammatory bowel diseases: A meta-analysis by Geng Z and Geng Q. (2022) Front. Surg. 9: 636791. doi: 10.3389/fsurg.2021.636791

**Incorrect Affiliation**

In the published article, there was an error in affiliation [1]. Instead of “Department of Orthopedics of Union Hospital, Tongji Medical College, Huazhong University of Science and Technology, Wuhan, China”, it should be “Department of Orthopaedics, Union Hospital, Tongji Medical College, Huazhong University of Science and Technology, Wuhan, China”.

The authors apologize for this error and state that this does not change the scientific conclusions of the article in any way. The original article has been updated.

