# Supplementary material for: Risk of Urinary Bladder Cancer in Patients With Inflammatory Bowel Diseases: A Meta-Analysis
Source: Front Surg. 2021 May 26;8:636791. doi: 10.3389/fsurg.2021.636791 (PMC8188732; doi:10.3389/fsurg.2021.636791)
Supplement: Supplementary file 1 [file Data_Sheet_1.docx]

**Supplementary file**

**Search strategies in detail**

**PubMed**

((((Inflammatory Bowel Diseases[Title/Abstract]) OR Inflammatory Bowel Disease[Title/Abstract]) OR Bowel Diseases, Inflammatory[Title/Abstract])) AND (((((((((((((((((((Urinary Bladder Neoplasms[Title/Abstract]) OR Neoplasm, Urinary Bladder[Title/Abstract]) OR Urinary Bladder Neoplasm[Title/Abstract]) OR Neoplasms, Bladder[Title/Abstract]) OR Bladder Neoplasms[Title/Abstract]) OR Bladder Neoplasm[Title/Abstract]) OR Neoplasm, Bladder[Title/Abstract]) OR Bladder Tumors[Title/Abstract]) OR Bladder Tumor[Title/Abstract]) OR Tumor, Bladder[Title/Abstract]) OR Tumors, Bladder[Title/Abstract]) OR Urinary Bladder Cancer[Title/Abstract]) OR Cancer, Urinary Bladder[Title/Abstract]) OR Malignant Tumor of Urinary Bladder[Title/Abstract]) OR Cancer of the Bladder[Title/Abstract]) OR Bladder Cancer[Title/Abstract]) OR Bladder Cancers[Title/Abstract]) OR Cancer, Bladder[Title/Abstract]) OR Cancer of Bladder[Title/Abstract]) 44 records

**Web of Science**

#1 TOPIC: (Inflammatory Bowel Diseases) OR TOPIC: (Inflammatory Bowel Disease) OR TOPIC: (Bowel Diseases, Inflammatory)

#2 TOPIC: (Urinary Bladder Neoplasms) OR TOPIC: (Neoplasm, Urinary Bladder) OR TOPIC: (Urinary Bladder Neoplasm) OR TOPIC: (Neoplasms, Bladder) OR TOPIC: (Bladder Neoplasms) OR TOPIC: (Bladder Neoplasm) OR TOPIC: (Neoplasm, Bladder) OR TOPIC: (Bladder Tumors) OR TOPIC: (Bladder Tumor) OR TOPIC: (Tumor, Bladder) OR TOPIC: (Tumors, Bladder) OR TOPIC: (Urinary Bladder Cancer) OR TOPIC: (Cancer, Urinary Bladder) OR TOPIC: (Malignant Tumor of Urinary Bladder) OR TOPIC: (Cancer of the Bladder) OR TOPIC: (Bladder Cancer) OR TOPIC: (Bladder Cancers) OR TOPIC: (Cancer, Bladder) OR TOPIC: (Cancer of Bladder)

#3 #1 and #2 115 records

**Cochrane and Embase**

#1 (Inflammatory Bowel Diseases or Inflammatory Bowel Disease or Bowel Diseases, Inflammatory).kw.

#2 (Urinary Bladder Neoplasms or Neoplasm, Urinary Bladder or Urinary Bladder Neoplasm or Neoplasms, Bladder or Bladder Neoplasms or Bladder Neoplasm or Neoplasm, Bladder or Bladder Tumors or Bladder Tumor or Tumor, Bladder or Tumors, Bladder or Urinary Bladder Cancer or Cancer, Urinary Bladder or Malignant Tumor of Urinary Bladder or Cancer of the Bladder or Bladder Cancer or Bladder Cancers or Cancer, Bladder or Cancer of Bladder).kw.

#3 #1 and #2 3 records
